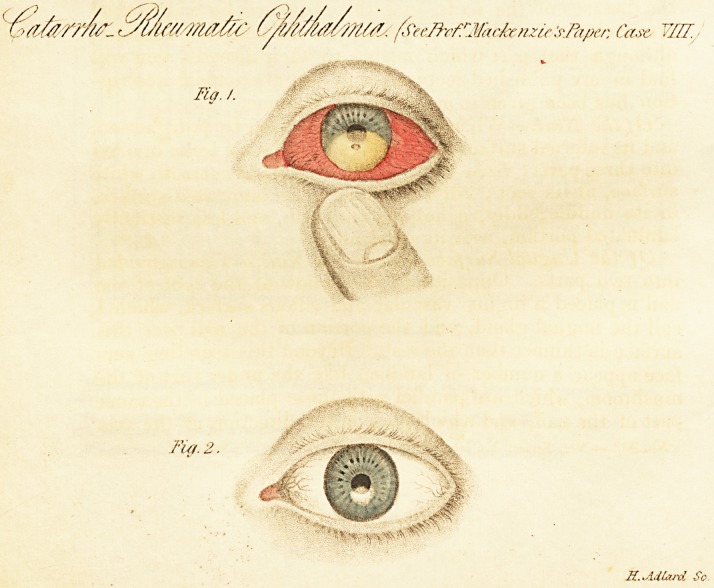# Practical Observations on Catarrho-Rheumatic Ophthalmia; with Cases

**Published:** 1827-04

**Authors:** William Mackenzie

**Affiliations:** Andersonian Professor of Anatomy and Surgery, and one of the Surgeons to the Glasgow Eye Infirmary.


H. Adlard Sc.

Engraved for the London Medical & Physical Journal, New Series, No. 10.

### CATARRHO-RHEUMATIC OPHTHALMIA.

*Practical Observations on Catarrho-Rheumatic Ophthalmia; with Cases.* By WILLIAM MACKENZIE, Andersonian Professor of Anatomy and Surgery, and one of the Surgeons to the GLASGOW EYE INFIRMARY.

[WITH AN ENGRAVING.]

SINCE the publication of my paper on Rheumatic Ophthalmia, I have received a communication from a surgeon in London, who for many years has devoted much of his attention to the diseases of the eye, in which he puts the following query—"What do you mean by *rheumatic* ophthalmia?" To which I should reply—

1. I mean simply inflammation of a fibrous tissue of the eye (the sclerotica), and of the surrounding parts of similar structure, excited by atmospheric changes.

2. I do not believe it to be an inflammation differing from common inflammation *in kind,* in consequence of the existence of what has been called the rheumatic habit or diathesis. When atmospheric influence produces catarrh, we never hear the occurrence referred to a mucous diathesis; nor, when pleuritis arises from the same cause, do we attribute the disease to a serous diathesis. The same exciting cause, affecting a fibrous, instead of a mucous or a serous membrane, produces a new train of symptoms, dependent not on the constitution of the person, but on the structure and functions of the part attacked.

3. Rheumatic ophthalmia frequently occurs in individuals who have never suffered from rheumatism in any other part of the body.

4. When rheumatism quits a joint and attacks the heart, which I have known to prove fatal, we say it is a metastasis from the former situation to the latter; but such a change of place I have never myself observed in regard to the eye. In all the cases of rheumatic sclerotitis which I have witnessed, the disease was primary, whether in rheumatic or in non-rheumatic subjects,—never metastatic.

5. I have taken on trust from BEER and WARDROP the term "rheumatic ophthalmia," lest I might shock the reader by a new name; yet "sclerotitis atmospherica" would be a truer appellation. This inflammation of the eye, however, resembles rheumatism in its exciting causes, its accompanying pain, its exacerbations, and its cure. It has been less generally recognised as a rheumatism, and some may even doubt that it is a rheumatism at all,—probably because it attacks a structure which is seen, a structure covered only by a thin semi-transparent membrane, and therefore exposed to direct examination: while the other seats of rheumatism, unlike this, are hid from our view by the whole thickness of the common integuments, and are the subjects, therefore, more of conjecture than of actual observation.

In two former communications (October 1826, and January 1827,) I described the pure catarrhal, and then the pure rheumatic ophthalmia, and illustrated the symptoms and treatment of these two diseases by cases, which I had treated publicly at the Glasgow Eye Infirmary, and which are recorded in the journals of that institution. I now proceed to the Catarrho-rheumatic Ophthalmia. In this disease, the conjunctiva and sclerotica are attacked simultaneously: the former membrane by blenorrhœal or catarrhal, and the latter by rheumatic inflammation. In this ophthalmia, then, the symptoms of the two diseases formerly described are conjoined.

One of your contemporaries has remarked, that these are *German* distinctions. I should say, they are natural distinctions—anatomical distinctions—physiological distinctions—practical distinctions, of the greatest importance to all who have eyes to be cured, and to all who intend to cure them.

When we examine the eye, we find its external covering formed by a mucous membrane, liable to profluvial or puro-mucous disease. Beneath it we find a fibrous texture, liable to rheumatic inflammation. In some ophthalmiæ we find the first membrane, in others the second membrane, affected, and in many instances both inflamed at once. We meet with many catarrhal cases of ophthalmia, in which no rheumatic disease is present; we meet with some cases (comparatively they are much fewer,) in which rheumatic sclerotitis exists, with redness, no doubt, of the conjunctiva, but without any catarrhal affection of this membrane. In a third set of cases, both tunics are not merely reddened, but the conjunctiva is affected with puro mucous inflammation, while the sclerotica is severely attacked by rheumatism.

The symptoms of these three sets of cases are strikingly marked; they cannot be overlooked by any practitioner of the least judgment and attention, to whom they are but once pointed out; they are characteristic, to the highest degree, of three different ophthalmiæ, requiring different modes of cure,—three ophthalmiæ, easily subdued if accurately distinguished and discriminately treated, but which, if confounded, are very apt to leave the eye permanently injured, or even deprived of sight. The practitioner who regards these as useless distinctions may, blundering on, light by chance on a treatment which, in an individual case or two, shall marvellously succeed; but to him who knows these distinctions, the treatment is determined and specific.

The German reader will find a section in Professor Beer's "Leitfaden," (vol. i. p. 302,) intitled *Idiopathische Catarrhalisch-rheumatisch Augenentzündung*. This is the only notice which I have seen taken of this disease by any author; but, instead of giving a description of catarrho-rheumatic ophthalmia, the learned Professor has entered into a disquisition on the causes and seat of acute and chronic rheumatism, and on the structure and connexions of the investing membrane of the eyelids and eyeball. The complicated affection which I am now about to describe, he passes over almost in silence; and it is to be regretted that in no other part of his most laboured and admirable work does he enter on the symptoms and treatment of catarrho-rheumatic ophthalmia. Yet this is one of the most common of the inflammatory diseases of the eye, and also one of the most severe and dangerous. In old persons especially, it is often the source of permanently diminished vision, and not unfrequently of entire loss of sight in the eye attacked.

### SYMPTOMS.

1. As both conjunctiva and sclerotica are affected in this disease, the symptoms are both more complicated, and also more various, than those of the unmixed conjunctivitis and sclerotitis, formerly described.

2. The sense of roughness, which is compared by the patient to the feeling of sand between the eyelids and eyeball, and the secretion of purulent mucus and purulent meibomian fluid, are sufficiently indicative of the part taken in this disease by the conjunctiva. The nocturnal accession of racking circumorbital pain marks the affection of the fibrous sclerotica, the surrounding periosteum, and the neighbouring temporal fascia.

3. In some cases of catarrho-rheumatic ophthalmia, the conjunctivitis is severe, the sclerotitis slight; but more frequently the sclerotitis is severe, the conjunctivitis not so considerable.

4. In this disease the conjunctiva and sclerotica are attacked simultaneously. Occasionally it happens in the course of pure rheumatic ophthalmia, that the patient, from some new exposure, becomes affected also with catarrhal conjunctivitis, as in the case of Mary Scott, page 44. More rarely does an attack of rheumatic sclerotitis supervene on catarrhal ophthalmia. But in catarrho-rheumatic ophthalmia, both membranes appear to be attacked at once, in consequence of the influence of one and the same exciting cause.

5. In this disease, the redness is evidently both conjunctival and sclerotic. Under the moveable network of the conjunctiva, we perceive the immoveable zonular inflammation of the sclerotica. In pure catarrhal ophthalmia, the sclerotica, no doubt, partakes in the inflammation of the contiguous tunic, but no paroxysms of rheumatic pain are present: the sclerotica suffers sympathetically, not primarily. In pure rheumatic ophthalmia, also, the conjunctiva is reddened, from contiguous sympathy with the structure which it invests, just as the skin is reddened over a joint suffering from acute rheumatism; but neither the conjunctiva in the one instance, nor the skin in the other, is the seat of the primary disease. Besides, in pure rheumatic ophthalmia, the conjunctiva betrays no marks of profluvial disease.

6. Chemosis, or inflammatory œdema of the sub-conjunctival cellular substance, is by no means an uncommon attendant on catarrho-rheumatic ophthalmia. When it does occur, it hides from our view the sclerotic redness.

7. The discharge from the conjunctiva in this disease is never profuse, and seldom opaque. It amounts, in general, rather to a mere increase of mucus, than a flow of pus.

8. The eyelids adhere together in the morning, from the inspissated meibomian secretion. Not unfrequently they are also externally red and swollen.

9. Considerable intolerance of light and epiphora attends this ophthalmia, in all its stages; but especially in those cases where the structure of the cornea is affected.

10. The conjunctival pain, which is compared to the feeling produced by sand between the eyelids and eyeball, is felt most in the morning, or when the eyelids are moved. The sclerotic pain is nocturnal, and observes the same periods of renewal, violence, and abatement, which I have noticed in my paper on Rheumatic Ophthalmia. The conjunctival pain is referred to the surface of the eye, and sometimes to the forehead. The sclerotic pain is circumorbital.

11. In this disease, the cornea is extremely apt to suffer from ulceration, and from effusion of pus between its lamellæ. Indeed, there is no ophthalmia to which adults are exposed, in which ulcer of the cornea and onyx are so frequent, as in the catarrho-rheumatic. If this disease is neglected for eight or ten days, and especially if the patient be far advanced in life, we almost uniformly meet with one or other, and not unfrequently with both, of these symptoms.

12. The ulcer is peculiar. It spreads over the surface, rarely penetrating deeply into the substance, of the cornea. It generally cicatrises without leaving any opaque speck, the cornea remaining merely irregular, as if part of it had been hacked off with the lancet; and of course vision, from imperfect refraction, is confused. Professor BEER and Mr. WARDROP have described this kind of ulcer as attendant on pure rheumatic ophthalmia, but I have never seen it except in catarrho-rheumatic cases. Professor Beer mentions that it originates in a phlyctenula, but I have never had an opportunity of seeing any appearance of this kind. If the case continues to be neglected, or if it be mistreated, this ulcer ceases to be superficial; the substance of the cornea is more deeply attacked, and opaque leucoma will be the result. (See Case V.)

13. Onyx, or effusion of pus between the lamellæ of the cornea, is the most alarming of all the symptoms of this ophthalmia. (See Fig. 1.) It generally commences at the lower edge of the cornea, in shape like the white spot at the root of the nails, convex on its upper edge, gradually increasing, mounting upwards, separating the lamellæ more and more between which it is effused, and greatly adding to the sufferings of the patient. It reaches not unfrequently to such a height as to implicate more than half of the cornea. The pus of an onyx in catarrho-rheumatic ophthalmia is very rarely absorbed. The cornea becomes ulcerated over the centre of the onyx, (as in Case VIII. Fig. 1;) the pus is evacuated; the ulcer penetrates through the posterior lamellæ of the cornea; the aqueous humour escapes; the iris falls forward into contact with the ulcerated cornea; in nine cases out of ten, these parts adhere together, and the result is partial or total staphyloma.

14. As the onyx goes on advancing, there is commonly also an effusion of lymph going on in the pupil: the pupil becomes, first of all, less vivid in its motions; the colour of the iris changes; the pupil becomes hazy, contracts as the onyx increases, and may at last be obliterated. (See Case VI.)

15. In some cases, the onyx is accompanied by hypopium, or effusion of pus into the anterior chamber. In other cases, the onyx bursts first into the anterior chamber; false hypopium is thus produced, and ultimately the cornea gives way.

16. If luckily the matter of an onyx be absorbed, albugo remains for a considerable time, but gradually diminishes, and may ultimately almost entirely disappear. If onyx is dispersed by the cornea giving way, leucoma is the result, and never entirely disappears. Staphyloma cannot result, unless the iris and cornea have become partially or totally adherent. Mr. Wardrop remarks, that partial staphyloma generally affects the inferior half of the cornea.* The reason is, that partial staphyloma is commonly the consequence of onyx, which in nine cases out of ten takes place at the lower edge of the cornea.

17. In catarrho-rheumatic ophthalmia, the pulse is generally quick and sharp; the tongue white, and mouth ill-tasted. The nocturnal pain completely prevents sleep, till about sun-rise. Catarrh sometimes attends, and adds to the febrile symptoms.

18. We generally find that the rheumatic symptoms yield first to treatment; the catarrhal continuing for some days longer. But in some cases I have observed the reverse: the circumorbital pain continuing in a slight degree after all the catarrhal symptoms were gone.

### CAUSES.

The causes of catarrho-rheumatic ophthalmia appear to be similar atmospheric influences to those formerly enumerated as giving rise to catarrhal, and rheumatic ophthalmiæ. Amongst the poor, the disease may in general be traced to cold, to which the patients have been exposed, particularly during the night, from deficient clothing and want of proper shelter. Like other inflammatory and rheumatic affections, it is more prevalent during north-easterly winds.

Professor Beer thought that cold draughts of air,† playing upon the eye, excited rheumatic ophthalmia; and that foul air‡ caused catarrhal ophthalmia. According to this view, air at once corrupted and impelled with force against the eye, especially when the head is covered with perspiration, will be the most likely cause of catarrho-rheumatic ophthalmia.

In 1805, at Riding-street Barracks, nearly twenty miles to the interior of Romney Marsh, the second battalion of the 52d Regiment appears to have suffered severely from catarrho-rheumatic ophthalmia. Dr. Vetch attributes the severity of the disease in that situation, and the intermittent form of some of the symptoms, to the influence of the marsh.§

That the discharge from the conjunctiva in catarrho-rheumatic ophthalmia, if applied to the conjunctiva of a

* Morbid Anatomy of the Eye, vol. i. p. 106.

† Eine kalte Zugluft.

‡ Ein zersetzer verdorbener Luftkreis.

§ See Vetch's Account of the Ophthalmia which has appeared in England since the Return of the British Army from Egypt, Lond. 1807, p. 20. Also Dr. A. T. Thomson's Remarks on Acute Rheumatism, in this Journal for February, p. 123.

healthy eye, will excite a puro-mucous conjunctivitis, is extremely probable, and is supported by such facts as I have recorded in Cases XII. and XIII. of a former communication.* That catarrho-rheumatic ophthalmia can arise from contagion, is extremely improbable. In such cases as that of Coleman and her child, (Case VII. of the present paper,) both patients had been exposed, we may conclude, to the same exciting cause; and, while the one caught catarrhal ophthalmia, the other was seized with the catarrho-rheumatic form of this disease.

Professor Beer mentions that catarrho-rheumatic ophthalmia sometimes occurs in children, and still more frequently in old persons, along with suppression of urine. But he seems to reject the conclusion of some, that this was any thing more than a mere coincidence; and he gives us no hope that diuretics would be peculiarly serviceable, even though they restored the secretion of urine.†

We meet with catarrho-rheumatic ophthalmia much more frequently in old persons than in the young or middle-aged.

### TREATMENT.

The successful treatment of this disease does not depend so much on any new remedies, as on a proper selection of some of the means formerly recommended, either for the catarrhal or for the rheumatic ophthalmia.

1. Venesection. This appears to be as necessary in the catarrho-rheumatic as in the pure rheumatic cases; and is attended by as remarkable relief to all the symptoms, especially to the circumorbital pain. According to the severity of the case, and the age and constitution of the patient, from ten to thirty ounces of blood may be taken from the arm; and the same quantity on the day following, if the symptoms are not greatly relieved.

2. Leeches to the temple are also highly useful, particularly when applied soon after venesection.

3. Scarification of the conjunctiva of the eyelids proves useful in cases of chemosis; but produces comparatively little effect, unless practised in the manner described at page 324, vol. lvi.

4. Calomel and Opium. The same good effects are derived from this combination in this ophthalmia, as in the pure rheumatic. The dose, and the length to which the calomel should be pushed, are the same. See page 41.

* Vol. lvi. p. 329.

† Beer's Leitfaden, vol. i. p. 310.

5. Opiate Frictions on the forehead and temple, about an hour before the expected attack of circumorbital pain.

6. Belladonna, so as to keep the pupil dilated.

7. Blisters behind the ear, or to the nape of the neck.

8. Purgatives; such as a brisk dose of calomel and jalap at the beginning, and a gentle laxative every morning during the course of the disease.

9. Sudorifics; such as Spiritus Mindereri, diluent drinks, the warm pediluvium, and a flannel under-dress.

10. Tonics; such as Cinchona and the Mineral Acids, in the chronic stage of the disease. Under these heads, I have nothing to add to what is stated at pages 41 and 42 of this volume, and at page 325 of vol. lvi.

11. Solution of Nitrate of Silver. As in the catarrhal, so in the catarrho-rheumatic ophthalmia, the solution of from two to four grains of nitrate of silver in one ounce of distilled water, dropped upon the conjunctiva once a-day, relieves the feeling of sand, and speedily removes the other symptoms of conjunctivitis. This application, however, has no effect on the sclerotic part of the disease; and I should conceive it a very dangerous mistake to trust to this remedy almost alone, as we may safely do in pure catarrhal ophthalmia, and to neglect the appropriate means for reducing the attendant inflammation of the sclerotica. See Mr. Melin's Report, in this Journal for September 1824.

12. Vinum Opii. Before the catarrhal part of this disease is subdued by the solution of nitrate of silver, this remedy rather aggravates the symptoms. After the conjunctivitis and the acute sclerotitis have yielded, it operates favourably, as in the chronic stage of the pure rheumatic ophthalmia; affording thus a good illustration of the remark of Boerhaave—"Nullum ego cognosco remedium nisi quod tempestivo usu fiat tale."

13. Collyrium Muriatis Hydrargyri, one grain to eight ounces, to be used milk-warm three or four times a-day.

14. Unguentum Præcipitati Rubri, smeared along the edges of the eyelids at bedtime. These I employ as part of the treatment suitable for the conjunctival part of the disease, according to the directions given at pages 325 and 326 of vol. lvi.

15. With respect to the treatment of onyx, I would recommend the lancet not to be used for evacuating the purulent fluid effused between the lamellæ of the cornea. In every case in which I have evacuated the matter with the lancet, partial or total staphyloma has been the result. In Ferrie's case, (Case VIII.) I left the matter to itself, and certainly no case could be more alarming in its progress, nor more unexpectedly happy in its results. I attributed the success which attended this case, in a great measure, to the sorbefacient influence of the calomel over the effusion into the pupil, —to the continued use of belladonna,—and to the gradual preparation of the cornea by nature for its giving way, and for its healing up; a preparation which would probably have been entirely defeated, had I ventured, as I had done in a number of previous cases, to open the onyx with the lancet.

Fig. 1, shows the onyx in this case, and the seat of the ulcer by which it was gradually evacuated. Fig. 2, shows the eye after recovery.

CASES.

Case I.—17th June, 1825.—Duncan M'Lean, aged forty-six. Since the 12th, severe pain in the right eye, eyebrow, and cheek, with redness of the conjunctiva and sclerotica, but chiefly of the latter. A superficial ulcer on the lower external part of the cornea. Adhesion of the eyelids in the morning.

Mitt. sang. e brachio ad ℥ xij. vel xv.—Cras mane adhib. Hirudines viij. ad temp. dextram.—℞. Subm. Hydr. gr. v.; Pulv. Jalapæ gr. xv. M. capiat q. p.—Belladonna ad palpebras dextras.

20th.—Pain of the eyebrow and cheek gone, and that of the eyeball much diminished; so that the leeches were not applied. Ulcer of the cornea nearly healed. Pupil about a medium size, but less than the other, and sluggish in its motions.

℞. Subm. Hydr. gr. ij.; Opii gr. ss. M. fiant tales doses vj. cap. j. m. et v.

22d.—Improves.

Vesicat. pone aurem dextram.—Sulphat. Magnes. ℥ jss.—Gtt. Sol. Nitr. Argent. ad oculum dextrum.

27th.—Mouth sore. Eye continues to improve.

29th.—Capiat Pulv. Cinchonæ ℈j. bis indies.

29th July.—Dismissed cured.

Case II.—4th August, 1825.—Robert Anderson, aged sixty-five. Catarrho-rheumatic ophthalmia of eight days' standing, with an ulcer on the lower internal part of the cornea of the left eye.

Hirudines viij. ad temp. sinistr.—℞. Subm. Hydr. gr. v.; Pulv. Jalap. gr. xv. M. capiat q. p.

5th.—Eye easier, and ulcer rather less.

Ung. Præc. rubri o. n. ad margines palpebrarum.

12th.—Inflammation rapidly declining, and ulcer contracting.

Gtt. Solutio Nitr. Argent. ad oculum sinistrum.

1st September.—Dismissed cured.

Case III.—11th November, 1825.—Margaret Young, aged twenty-three. Scattered redness of the left conjunctiva, with a superficial ulcer of the cornea. Pain round the orbit, increased when she is warm in bed. Adhesion of the eyelids in the morning.

Mitt. sang. e brachio ad ℥ xv.—℞. Subm. Hydr. gr. v.; Pulv. Jalapæ gr. xv. M. capiat q. p.

16th.—Symptoms much abated.

Utatur pro collyrio Sol. Mur. Hydr.

19th December.—Has been ill with cold since last report. The symptoms are all much increased. Pulse ninety-six, sharp.

Repetatur V.S.—Sulph. Magnes. ℥iss.

27th January, 1826.—Dismissed cured.

Case IV.—28th December, 1825.—Euphemia Wilson, aged twenty-six. Conjunctivitis catarrhalis of the right eye, of three weeks' standing; at first attended with considerable rheumatic pain of the head and cheek. Eyelids swollen.

Scarif. facies interna palpebr. infer. dextram.—Ung. Præc. rubri ad marg. palpebrarum o. n.

30th.—Gtt. Solutio Nitr. Argent.—Collyr. Mur. Hydr.

2d January, 1826.—Much improved.

30th.—Dismissed cured.

Case V.—20th January, 1826.—Agnes Sharp, aged sixty-six. Has suffered severely from catarrho-rheumatic ophthalmia in both eyes, four months ago. The greater part of the right cornea is leucomatous; the left appears to have been in the same state, but is now ulcerated, and the conjunctiva much inflamed. The left eye is very painful, as well as the upper part of the head and the left temple; left eyelids œdematous.

Extr. Belladonnæ ad palpebr. sinistr.—℞. Subm. Hydr. gr. ij.; Opii gr. j. M. fiant tales doses vj. cap. j. o. n.—Fricetur regio supraorbitalis Tinct. Opii.

22d.—Much relieved.—Vinum Opii.—Cont. Pulv. et Fricatio.

25th.—Ulcer of the left cornea cicatrised.

27th.—Eyes easy.

Omitt^r Pulv.—Sulphatis Magnesiæ ℥ j.—Cont^r Vinum Opi.

30th.—Inflammatory symptoms gone; but there seems to be no possibility of restoring vision by any species of operation. Dismissed relieved.

Case VI.—3d May, 1826.—Andrew Bain, aged seventy-six. Lost the sight of the right eye when a child, by small-pox. Catarrho-rheumatic ophthalmia, of eight weeks' standing. The centre of the left cornea has been in a state of ulceration, but is now cicatrised: the whole of the left cornea is nebulous; vision very obscure; supraorbital and temporal pain very much gone.

Vinum Opii.—Belladonna ad palpebras.—Oleum Ricini ℥ j.

5th.—Pulv. Doveri gr. x. h. s.—Cont^r Belladonnæ et Vinum Opii.

8th.—Sulphatis Magnesiæ ℥ j.—Ung. Præc. rubri o. n.—Cont^r Vinum Opii, Beladonnæ, et Pulv. Doveri.

10th.—Left cornea beginning to clear.

15th.—No pain, and can open the eye better, cornea clearing, but vision not returning; bowels confined.

Pil. Colocynth. pro re nata.—Cont^r Vinum Opii et Belladonnæ.—Omitt^r Pulv. Doveri.

24th.—The pupil, which is now coming into view in consequence of the clearing of the cornea, is exceedingly contracted.

℞. Subm. Hydr. gr. j.; Opii gr. ss. M. fiant tales doses xii. capiat j. m. et v. — Contr alia.

31st.—The cornea is now so clear as to permit the pupil to be seen: almost obliterated, and filled with an opaque effusion.

7th June.—Contr Pulv. Subm. Hydr. et Opii, h. s.

14th.—Cornea still clearer, and he begins to see better.

19th.—Powders purge.

Opii gr. j. o. n.—Contr Pulv. m. et v.

5th July.—Cornea still clearing, but little further improvement in vision.

℞. Subm. Hydr. gr. ij.; Opii gr. j. M. fiant tales doses xij. capiat j. o. n.—Omittr Pulveres priores.

7th.—Bowels bound.—Pil. Aleot. pro re nata.

10th.—Finds his vision improving, so that he was able to discern Knox's monument last night.

21st.—Purging and pain in the bowels.

Omittr Pulv.—Capiat h. s. Opii gr. j.—Fricetur regio circumorbitalis Ung. Hydr. Camphorato o. n.—Contr Vinum Opii et Belladonna.

28th.—Bowels regular.

Gtt. Vin. Opii c. Stramonio indies.—Contr Fricatio c. Ung. Hydr. Camphor.

25th September.—Has ceased to attend. Dismissed relieved.

Case VII.—5th May, 1826.—Margaret Coleman, aged twenty. Since the 1st, severe catarrho-rheumatic ophthalmia of both eyes, commencing in the left, which is still the most affected. Several small open pustules round the edge of the corneæ, particularly of the left; nocturnal pain from five P.M. till five A.M., worst about midnight; feeling of sand, and pulsating pain in both eyes, with rheumatic pain affecting the supraorbital and infraorbital regions; tongue dry; thirst; pulse eighty-four; adhesion of the eyelids in the morning. Thinks she got this complaint from her child, aged three, who has been affected with catarrhal ophthalmia.

Mitt. sanguis e brachio ad ℥ xij. vel xv., et repetatur V.S. cras, si opus fuerit.—℞. Subm. Hydr. gr. xij.; Opii gr. vj.; Pulv. Glyc. Glab. ℈j. M. divide in pulv. vj. capiat j. o. n.—Fricetur pars capitis dolens Tinct. Opii, m. et v. præsertim ante doloris superventionem.—Collyr. Mur. Hydrarg.

8th.—Did not send for the Collyrium nor Tincturæ Opii.

Gtt. Sol. Nitr. Argenti.—Vesicat. ad nucham, et postea Ung. Resinos.

10th.—Symptoms considerably abated.

Contr Pulveres et alia.

12th.—Eyes greatly better; no nocturnal pain, but complains of headache in the course of the day; her mouth is affected.

Omittr Pulv. Subm. Hydr. et Opii.—Sulphatis Magnesiæ ℥ jss.

15th.—Symptoms gone; mouth still sore.

Case VIII.—22d May, 1826.—John Ferrie, aged forty-seven. About three weeks ago became affected with catarrho-rheumatic ophthalmia of the left eye. For eight days past, he has had severe orbital pain during the night. Onyx of the cornea, extending from its lower edge to over the pupil; an ulcer over the middle of the onyx. Much vascularity of the conjunctiva and sclerotica.

Gtt. Vin. Opii.—Belladonnæ ad palpebr.—℞. Subm. Hydr. gr. ij.; Opii gr. j. M. fiant tales doses vj. capiat j. o. n.—Fricentur partes dolentes Tinct. Opii, o. n.—Pediluvium tepidum h. s.

24th.—Feels the eye better, although there is not much evident change in its appearance. Iris discoloured, and an effusion into the pupil.

Contr Pulv. Subm. Hydr. et Opii, m. et v.—Vesicat. ad nuch.—Contr alia.

27th.—Onyx increasing; mouth affected.

Omittr Pulv. matutin.—Hirudines viij. ad temp. sinistr.

31st.—Pupil still contracting.

2d June.—Upper part of the cornea more nebulous; feels the eye more uneasy.

Gtt. Solutio Nitr. Argent.

5th.—The exterior laminæ of the cornea have given way, and discharged a considerable quantity of matter from the onyx. Pupil still more contracted. A feeling of sand in the eye.

℞. Extr. Belladonnæ ʒss; Aquæ ℥ vj. solve, et cola, pro collyrio.

7th.—Vesicat. pone aurem sinistram.

9th.—The aqueous humour has evacuated itself since the 7th, and the iris fallen forward into contact with the cornea. Matter of the onyx almost entirely gone. Says he sees a little better.

Contr Pulv. Submur. Hydr. et Opii.

12th.—Pupil in contact with the cornea clearer, and vision more distinct.—Cont. medicamenta.

14th.—A little aqueous humour between the upper part of the iris and cornea; ulcer of the cornea covered with lymph; all the pus gone.—Cont. Belladonnæ et alia.

26th.—Pupil considerably enlarged, and clear; more aqueous humour between the iris and cornea.—Cont. Solut. Belladonnæ.

30th July.—Pupil clear, of considerable size, and vision good. A minute adhesion between the leucoma and the lower edge of the pupil, not observed when the eye is viewed in front. A slight speck continues in the seat of the ulcer. Dismissed cured.

*Spreull's-court, Glasgow; Feb.* 1827.

## Figures and Tables

**Fig. 1. Fig. 2. f1:**